# A Method to Determine BeiDou GEO/IGSO Orbital Maneuver Time Periods

**DOI:** 10.3390/s19122675

**Published:** 2019-06-13

**Authors:** Zhiwei Qin, Guanwen Huang, Qin Zhang, Le Wang, Xingyuan Yan, Yanchao Kang, Xiaolei Wang, Shichao Xie

**Affiliations:** 1 College of Geology Engineering and Geomantic, Chang’an University, 126 Yanta Road, Xi’an 710054, China; gnssorbitqzw@163.com or gnssorbitqzw@chd.edu.cn (Z.Q.); zhangqinle@263.net.cn (Q.Z.); rexlele@163.com (L.W.); yanxydice@163.com (X.Y.); niuroumian@163.com (Y.K.); wonderwall@chd.edu.cn (S.X.); 2 School of Earth Sciences and Engineering, Hohai University, 1 Xikang Road, Nanjing 210098, China; gnssrwxl@126.com

**Keywords:** GEO/IGSO, period detection, orbital maneuver, time periods, orbit prediction

## Abstract

Because there are different types of BeiDou constellations with participating geostationary orbit (GEO) and inclined geosynchronous orbit (IGSO) satellites, the maneuvering frequency of BeiDou satellites is higher than that of other navigation systems. The satellite orbital maneuvers lead to orbital parameter failure for several hours from broadcast ephemeris. Due to the missing initial orbit, the maneuvering thrust, and the period of orbital maneuvering, the orbit products of maneuvering satellites cannot be provided by the International Global Navigation Satellite System (GNSS) Service (IGS) and International GNSS Monitoring and Assessment System (iGMAS). In addition, the period of unhealthy status and the orbital parameters of maneuvering satellites in broadcast ephemeris are unreliable, making the detection of orbital maneuver periods more difficult. Here, we develop a method to detect orbital maneuver periods involving two key steps. The first step is orbit prediction of maneuvering satellites based on precise orbit products. The second step is time period detection of orbit maneuvering. The start time detection factor is calculated by backward prediction orbit and pseudo-range observations, and the end time detection factor is calculated by forward prediction orbit and pseudo-range observations. Data of stations from the Multi-GNSS Experiment (MGEX) and iGMAS were analyzed. The results show that the period of orbit maneuvering could be detected accurately for BeiDou GEO and IGSO satellites. In addition, the orbital maneuver period of other GNSS medium Earth orbit (MEO) satellites could also be determined by this method. The results of period detection for orbit maneuvering provide important reference information for precision orbit and clock offset determination during satellite maneuvers.

## 1. Introduction

The development of the BeiDou Navigation Satellite System (BDS) of China was planned in three steps: an experimental system, a regional system, and a global system [1]. The satellites constellation of BDS consists of a geostationary orbits (GEO: C01, C02, C03, C04, C05), inclined geosynchronous orbits (IGSO: C06, C07, C08, C09, C10, C13), and medium Earth orbits (MEO: C11, C12, C14). Since 27 December 2012, GEO and IGSO satellites have played an important role in regional BDS applications across the Asia-Pacific region [2,3,4,5,6]. However, they bring great challenges to orbit determination, especially for GEO satellites with the demand of geostationary orbit. In order to maintain the geosynchronous characteristics of the GEO and IGSO satellites, their frequency of orbit maneuvering is higher than MEO satellites [7,8,9,10]. During orbit maneuvers, the dynamic models for determining the orbit are different during maneuvering and non-maneuvering periods. The kinetic model is unknown because of the secret maneuvering thrust during the orbit maneuvering period, but the orbit of the non-maneuvering period can be determined by the normal kinetic model. In addition, the status vectors estimated of the perturbation parameter including the position, velocity, and solar radiation pressure for the satellite are different before the start time and end time of orbital maneuvers, which means the same orbital parameters cannot be used for orbit integrations. Because the period of orbit maneuvering is unknown, the precise orbits of satellites cannot be determined during the non-maneuvering period. In order to adjust the strategy of precise orbit determination, the initial orbit and maneuver period must be determined. Therefore, it is necessary to develop an approach for detecting satellite orbit maneuvering periods with good time resolution.

Recently, Yan et al. presented a detection method to determine orbital maneuvers with the restored orbit calculated by broadcast ephemeris of BDS [11]. Ye et al. determined orbit maneuvering by the root mean square of the orbit from the BeiDou satellites [12]. Cui et al. detected orbital maneuvers by the orbit residual, which requires the known orbital maneuver dynamic models [13]. Su et al. used the mechanical energy difference of unit mass between space target and spacecraft to detect the orbital maneuver by the simulated data, and the effect of their method was restricted by the number of stations [14]. Du et al. determined orbit maneuvering by the orbit measurement data of the China Area Positioning System (CAPS), but the data is not available for the common users [15]. Sciré et al. analyzed the spatial debris orbit determination algorithm using space-based optical observation data [16]. Huang et al. detected the start time of orbital maneuvers by using single point positioning (SPP) technology and residual values of a pseudo-range [17,18].

Although some research has been done to detect the orbit maneuvering of Global Navigation Satellite System (GNSS) satellites, prior complex mechanical models and additional measurement data are both restricted for the common user, and the time period of orbit maneuvering has not been determined. Moreover, the maneuver period and the initial orbit could not be detected by publicly available observation data. In order to solve the above problems, this paper proposes a backward integration method to calculate the positions of maneuvering satellites by measured orbits on the day after orbital maneuvering, and subsequently calculate the residual value of the pseudo-range to determine the period of orbital maneuvers.

## 2. Theory of Orbital Maneuver Period Detection

The orbits of satellites change significantly before and after maneuvering. Considering that pseudo-range observations and the satellite clock are not influenced by maneuvering, the orbital error will increase gradually after the satellite orbit is switched. Based on this, the time period of a satellite orbit maneuver can be determined by using pseudo-range observations and the positions of the station and the satellite. In our previous research, we provided detecting methods for BDS satellite orbital maneuvers [17,18]. However, the orbit after maneuvering is lost from broadcast ephemeris. The precise products of orbit maneuvering satellites are also lost from the International GNSS Service (IGS) or International GNSS Monitoring and Assessment System (iGMAS), which causes difficulty in determining the time period of orbit maneuvering. Based on this, the time period of satellite orbit maneuvering is determined using predicted orbits by precise products on the day after orbital maneuvers. The orbit before maneuvering is predicted by precise products on the day before orbital maneuvers, and it also can be replaced by broadcast ephemeris or ultra-rapid orbit products at 00:00.

### 2.1. Analysis of Satellite Orbit Maneuvers

During normal operation of the satellite, in order to correct the perturbation from the space environment, orbit maneuvering is necessary to adjust the position of the satellite. When the satellite is maneuvering, the working time of the propulsion system is much less than the operating period of the satellite. Therefore, satellite orbits are adjusted by a pulsing method. The pulsing thrust makes the satellite velocity change suddenly, but the variation of radial distance is small [19]. The common user cannot obtain information of the maneuvering thrust because of secrecy. However, changes in the radial direction can easily be reflected by pseudo-observations of stations. The effects of orbital maneuvers in the radial direction are discussed below.

**Figure 1:**
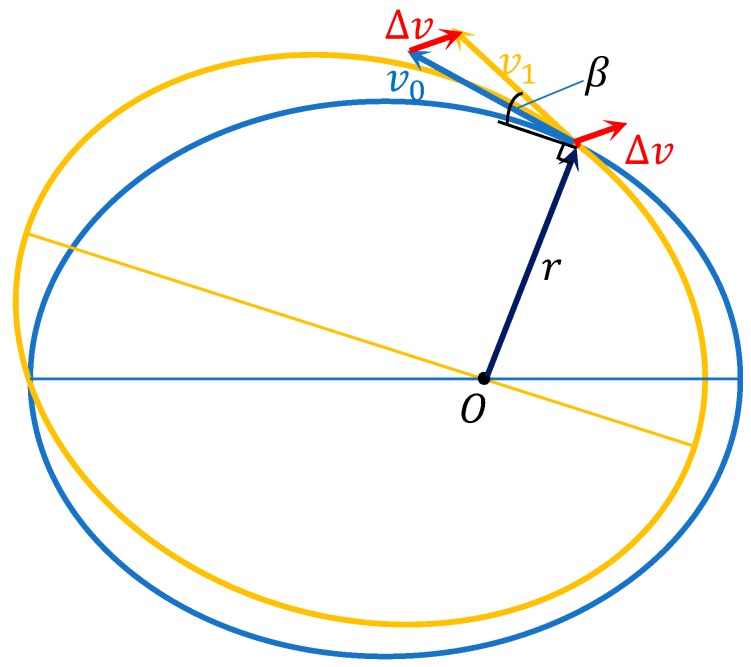
Schematic diagram of satellite orbit maneuvering.

In Figure 1, the blue track is the orbit of the satellite before maneuvering and the yellow track is the orbit after maneuvering, and O is the center of the Earth. At time $t_{0}$, r denote the position vector of the satellite, and $v_{0}$ is the velocity of the satellite. At that time, Δv denote the orbit maneuvering increasing the instantaneous velocity of the satellite; $v_{1}$ is the velocity of the satellite after the orbit thrust action; and β is the complementary angle of the angle between r and $v_{1}$:

(1) $$\dot{r_{1}}=v_{1}sin\beta =\left(v_{0}+\Delta v\right)sin\beta =v_{0}sin\beta +\Delta vsin\beta$$

(2) Δd=ΔvsinβΔt

where $v_{0}$ is the velocity of the satellite at time $t_{0}$; Δv is the orbit maneuvering increasing the instantaneous velocity of the satellite; $v_{1}$ is the velocity of the satellite after the orbit thrust action; β is the complementary angle of the angle between r and $v_{1}$; and $\dot{r_{1}}$ is the velocity vector of the satellite in the radial direction after orbit maneuvering, that is accumulating during a time period of Δt and Δd denotes the variation of distance due to the orbital maneuvers. The results of analyzing the effect of increased velocity of orbit maneuvering on radial distance are as follows.

The satellite orbital maneuvers are divided into two situations: one is the orbital adjustment to make the satellite go into the designed orbit, and the other is the orbit maneuvering, which ensures that the satellite runs in the normal orbit. The maneuvering thrust of the latter is less than that of the former, and the increased velocity of orbit maneuvering and the value of β are small. In addition, the efficiency of the orbit maneuvering thrust in the tangential direction is twice the radial direction. Efficiency is estimated using the amount of kinetic energy consumed by the satellite propulsion systems, which depends on the eccentricity of different satellites constellation. The efficiency of the radial orbit maneuvering thrust to correct the mean longitude of the satellite is less, and the increased velocity is 2.66 m per second to adjust 0.1°. It only needs to be 0.28 m per second to achieve it in the tangential direction, the time it takes to obtain the longitude change depends on the orbital semimajor axis (the derivation is not described in this paper, and is proved in [19]). In order to extend the service time of satellites, save fuel used by the propulsion systems, and raise efficiency for orbit maneuvering, the orbit maneuver’s increased velocity in the tangential direction is greater than the radial direction, which reduces the values of Δv and β. Therefore, the variation of distance in the radial direction and the variation of pseudo-range observations are small due to the orbital maneuvers. It is difficult to determine the start time of orbital maneuvers by using the satellite orbit before maneuvering and, similarly, to detect the end time after maneuvering. 

### 2.2. Detection Factor of Orbital Maneuver Time Period

**Figure 2:**
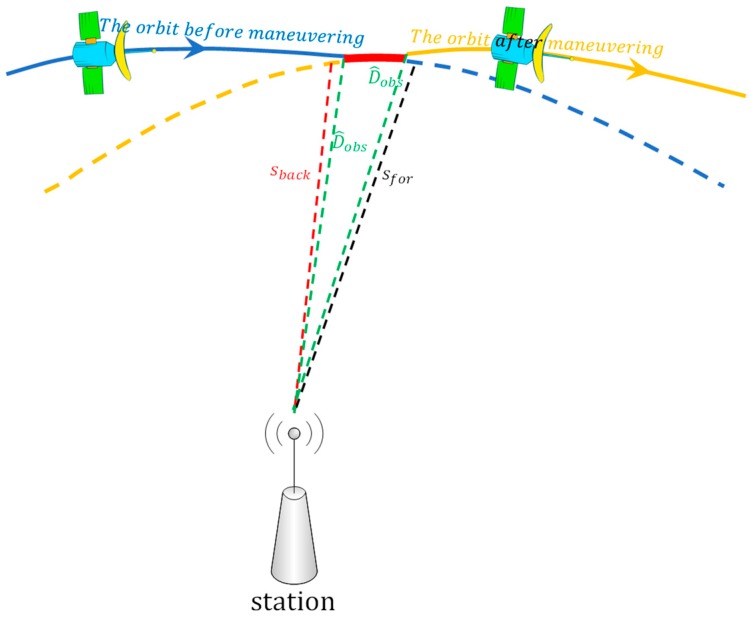
Schematic scheme of orbital maneuver detection.

In order to find a method to determine the period of orbit maneuvering and combine with the above analysis results, a schematic scheme of orbital maneuvering was used (Figure 2).

In Figure 2, the solid blue line denotes the orbit of the satellite before orbit maneuvering; the solid yellow line denotes the orbit of the satellite after orbit maneuvering; the dotted blue line denotes the forward predicted orbit after orbit maneuvering; the dotted yellow line denotes the backward predicted orbit before orbit maneuvering; and the solid red line denotes the orbit during orbit maneuvering. The dotted green line denotes the measured distance between station and satellite from the receiver; the dotted red line is the calculated distance using the station coordinates and the predicted orbits by precise products on the day after orbital maneuvers; and the dotted black line is calculated distance using the station coordinates and predicted orbits by precise products on the day before orbital maneuvers. The intersection point of the solid blue and red lines is the start time and the intersection point of the solid red and yellow lines is the end time for orbit maneuvering:

(3) $${\hat{D}}_{obs}=D_{obs}+c\delta t^{j}-c\delta t_{i}+\delta I+\delta T$$

where $D_{obs}$ is the pseudo-range observation; c is the velocity of light; $\delta t^{j}$ is the clock offset of the satellite; $\delta t_{i}$ is the clock offset of the receiver; δI is the ionospheric delay correction; δT is the tropospheric delay correction; and ${\hat{D}}_{obs}$ is the value of pseudo-range observation reduced the clock offset of the satellite and receiver, the ionospheric delay correction and the tropospheric delay correction.

It needs to be emphasized that in order to correctly calculate the clock offset of the receiver, it needs to be estimated through the least squares and robust methods [17,18]:

(4) $$S_{for}=\sqrt{{\left(X_{for}^{j}-X_{i}\right)}^{2}+{\left(Y_{for}^{j}-Y_{i}\right)}^{2}+{\left(Z_{for}^{j}-Z_{i}\right)}^{2}}$$

where $X_{i},Y_{i},Z_{i}$ are the station coordinates; $X_{for}^{j},Y_{for}^{j},Z_{for}^{j}$ are the spatial coordinates of satellite j, which is calculated by the forward predicted orbit by the precise products on the day after orbital maneuvers; and $S_{for}$ is the distance calculated between the satellite and the station.

(5) $$S_{back}=\sqrt{{\left(X_{back}^{j}-X_{i}\right)}^{2}+{\left(Y_{back}^{j}-Y_{i}\right)}^{2}+{\left(Z_{back}^{j}-Z_{i}\right)}^{2}}$$

where $X_{back}^{j},Y_{back}^{j},Z_{back}^{j}$ are the coordinates of the satellite, calculated by the backward predicted orbit by the precise products on the day before orbital maneuvers; and $S_{back}$ is the distance calculated between the satellite and the station.

Whether or not orbital maneuvers occur, ${\hat{D}}_{obs}$ is closer to the real distance between the satellite and the station, which cannot be influenced by orbit maneuvering. However, from Figure 2 we know that $S_{back}$ is inaccurate, with increasing errors before the start time of orbital maneuvers, because of the incorrect backward predicted orbit (dotted yellow line in Figure 2). Thus, the start time of orbit maneuvering can be detected by using the difference between $S_{back}$ and ${\hat{D}}_{obs}$. Similarly, the end time can be determined by using the difference between $S_{for}$ and ${\hat{D}}_{obs}$:

(6) $$V_{back}=\left|S_{back}-{\hat{D}}_{obs}\right|$$

where $V_{back}$ is the absolute value of the difference between $S_{back}$ and ${\hat{D}}_{obs}$:

(7) $$V_{for}=\left|S_{for}-{\hat{D}}_{obs}\right|$$

where $V_{for}$ is the absolute value of the difference between $S_{for}$ and ${\hat{D}}_{obs}$.

The start and end time discrimination factor $L_{start}$ and $L_{end}$ of the satellite orbit maneuver is defined by:

(8) $$L_{start}=D_{back}-T_{Max}$$

(9) $$L_{end}=D_{for}-T_{Max}$$

where $T_{Max}$ is the empirical threshold of $V_{back}$ and $V_{for}$.

The empirical threshold $T_{Max}$ of the satellite is given in advance, which is key to detecting the period of orbital maneuvers. Considering that the absolute value of the difference between S and ${\hat{D}}_{obs}$ follows normal distribution, the values of $V_{back}$ and $V_{for}$ in the normal condition of satellites are all in the interval of 99.73% (3σ) confidence coefficient. That is, if $V_{back}$ and $V_{for}$ are out of the corresponding confidence interval, it is considered to be abnormal. Thus, $T_{Max}$ for satellites is given by the upper limit of the confidence interval of the difference between S and ${\hat{D}}_{obs}$ for the 99.73% confidence coefficient, which uses data from 2017. When $L_{start}$ is greater than 0 and keeps a sustained decreasing trend in a period, a 5 min time period (10 epochs) is chosen for this study. The time of the last epoch when $L_{start}$ is greater than 0 is the start time of orbit maneuvering. Similarly, when $L_{start}$ is greater than 0 and keeps a sustained growth trend in a period, the end time of orbit maneuvering is the time of the first epoch when $L_{end}$ is greater than 0. The orbital maneuver time period can be determined by the start time and end time detected by the start time and end time factors.

### 2.3. Method for Predicting the Orbit

The spatial coordinates of the station, pseudo-range observations, and satellite position are needed to calculate the start time and end time factors. Once the orbit maneuver occurs, the orbital parameters in broadcast ephemeris are inaccurate, with increasing errors for several hours (generally about 7–8 h for BDS) and the precise orbit products of the maneuvering satellite are lost from IGS and iGMAS, causing difficulty in determining the time period of orbital maneuvers.

In order to determine the start time of orbital maneuvers, the satellite position is calculated by the backward predicted orbit, which can be predicted from the precise orbit products on the day after orbit maneuvering. The orbital parameters of satellite after orbit maneuvering are consistent with the day after orbit maneuvering, which can calculate the correct position of satellite. Furthermore, the orbital parameters of satellite before orbit maneuvering are consistent with the day before orbit maneuvering, which can calculate the correct position of satellite. The forward and backward predicted orbit can be determined by the precise orbit products using the following formula:

(10) $$\left(r_{t},{\dot{r}}_{t}\right)=g\left(t,r_{0},{\dot{r}}_{0},q\right)$$

where $r_{0}$ is the initial position vector; ${\dot{r}}_{0}$ is the initial velocity vector; q is the status vector of the perturbation parameter for the satellite; and *t* is the time vector. The perturbation parameters of the satellite are calculated by orbit fitting by the measured orbit. The orbit is predicted by orbit integrations with parameters, including $r_{0}$, ${\dot{r}}_{0}$, and the perturbation parameters. Specific mathematical models of orbit fitting and integration can be found in [20]. 

To detect the end time of orbital maneuvers, the satellite position is calculated by the forward predicted orbit. The orbit before maneuvering is predicted by precise products on the day before orbital maneuvers, and can also be replaced by broadcast ephemeris or ultra-rapid orbit products at 00:00:00. 

In this study, we used precise orbit products from the Chang’an University BeiDou Analysis and Service Center (CHD) to predict the backward and forward orbits for orbit maneuvering of BeiDou satellites.

## 3. Experimental Validation

In order to validate the proposed method for detecting the period of orbit maneuvering for GEO and IGSO satellites, the results of orbit maneuvering detection for the period are analyzed. 

### 3.1. Data Description

**Figure 3:**
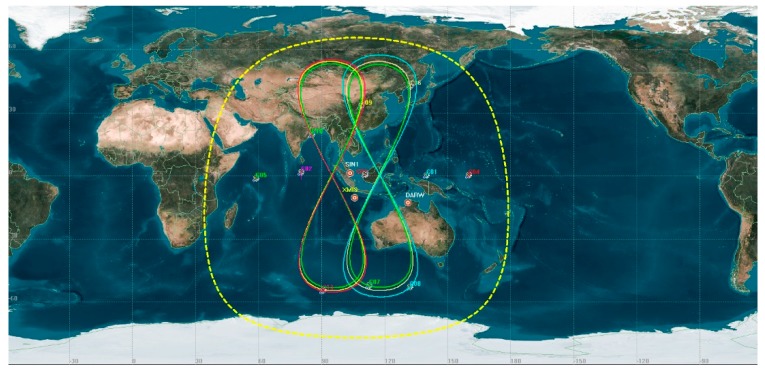
Satellite trajectories on the ground and station locations. Red circles denote selected station locations. Satellites with distributions of trajectories in dotted yellow circle can be monitored by the XMIS (Christmas Island) station.

Data from the Multi-GNSS Experiment (MGEX) stations XMIS (located on Christmas Island), DARW (in Darwin), and SIN1 (in Singapore) with a 30 s sampling period were selected to analyze the experimental results of orbit maneuvering detection of BeiDou GEO and IGSO satellites, which combine predicted orbits by using the precise orbit products from CHD. The station coordinates were obtained from the IGS Solution Independent Exchange (SINEX) product. The distributions of trajectories on station XMIS and DARW are shown in Figure 3. 

Thresholds of orbital maneuver detection are shown for XMIS station in Table 1, calculated by normal observations and the predicted orbit in 2017.

### 3.2. Orbital Maneuver Period Detection

#### 3.2.1. Orbital Maneuver Period Detection for GEO Satellites

**Figure 4:**
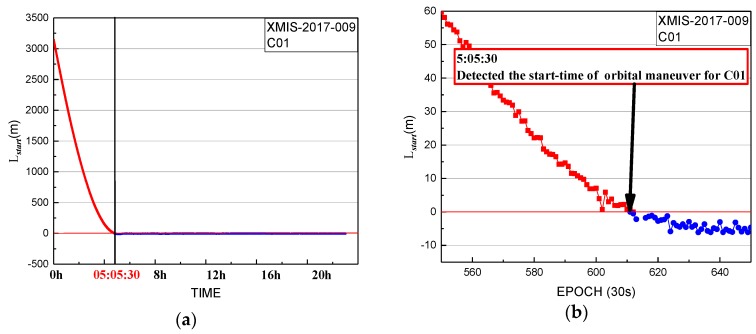
Start time factor of XMIS station for C01 on 9 January 2017: (**b**) detailed information from graph in (**a**).

The period of orbit maneuvering was determined for C01 on 9 January 2017. The performance of the start time factor series for XMIS is shown in Figure 4.

In Figure 4, the start time factor series shows a sustained decreasing trend over about 5 h with values greater than 0 until 5:05:30. The start time of orbit maneuvering of C01 determined by the start time factor on 9 January 2017 is 5:05:30.

**Figure 5:**
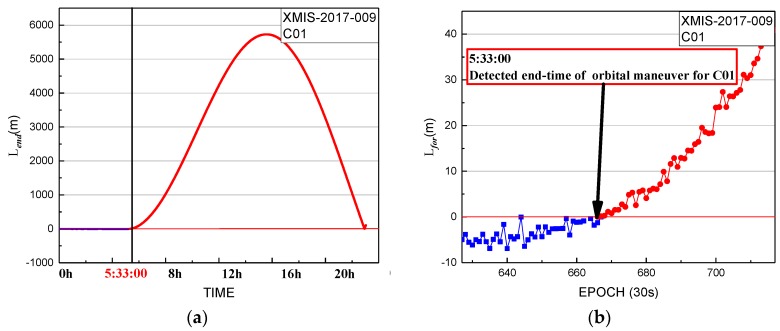
End time factor of XMIS station for C01 on 9 January 2017: (**b**) detailed information from graph in (**a**).

The performance of the end time factor series for XMIS is shown in Figure 5.

In Figure 5, the end time factor series shows a sustained growth trend with values greater than 0 until the start at 5:33:00. The end time of the orbital maneuver of C01 detected by the end time factor on 9 January 2017 is 5:33:00.

Thus, the period of orbit maneuvering for C01 can be determined by the start time and end time factors. In order to validate the results, the health status information of the satellite from broadcast ephemeris and to decide whether the satellite was included in the final precise orbit products of iGMAS and the German Research Centre for Geosciences (GFZ), GFZ was used as a reference.

**Figure 6:**
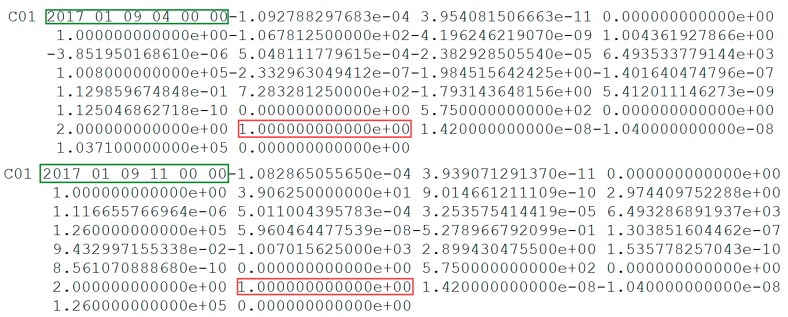
Broadcast ephemeris of C01. The red box highlights the unhealthy status marked as 1. The blue box highlights the healthy status marked as 0. Note that the data are not displayed between 4:00:00 and 11:00:00 to limit the height of the figure and the healthy statuses are all marked as 1 in this period.

**Figure 7:**
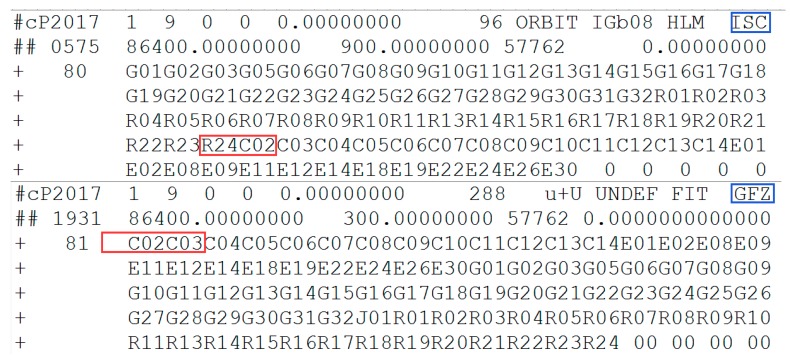
Information of precise orbit products from International Global Navigation Satellite System (GNSS) Monitoring and Assessment System (iGMAS) and German Research Centre for Geosciences (GFZ) on 9 January 2017. The red box highlights the satellite removed from the header of precise orbit products. The blue box marks the abbreviation of the organization providing the products.

The parameters of C01 from broadcast ephemeris and the header information of precise orbit products on 9 January 2017 are shown in Figure 6 and Figure 7, respectively.

In Figure 6, the status of C01 is marked as unhealthy from 04:00 to 11:00. The period of orbital maneuver determined for C01 is 5:05:30 to 5:33:00 (between 4:00:00 and 11:00:00). As is known, after orbit maneuvering, the orbital parameters of the satellite are different from the previous parameters. Once the orbit is maneuvered, the precise orbit of C01 cannot be determined because orbit maneuvering leads to failure of the kinetic empirical parameters. The precise orbit products did not include the C01 in Figure 7, which is secondary proof of the orbit maneuvering for C01.

**Figure 8:**
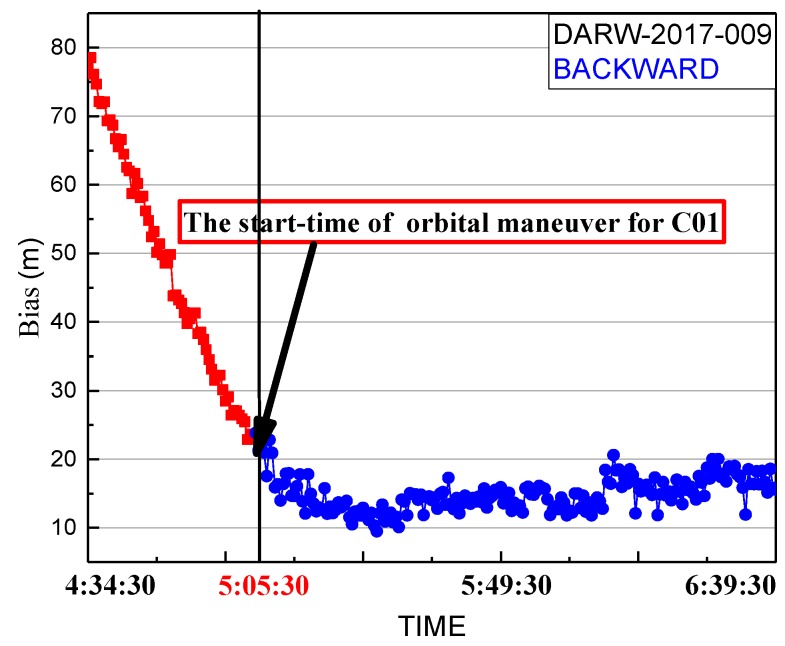
Spatial bias of pseudo-range single point positioning (SPP) coordinates on 9 January 2017. Coordinates are calculated by backward predicted orbit using the precise products on the day after orbital maneuvers. The red line is the series before the start time determined of maneuvering. The blue line is the series after the start time determined of maneuvering.

Although the period of unhealthy marks of C01 from broadcast ephemeris include the determined period of orbital maneuver and the precise products of iGMAS and GFZ removing the C01 satellite, the orbit maneuvering period determined by this method needs to be verified that it is near the real period of orbit maneuvering. Therefore, the bias between pseudo-range SPP coordinates of DARW station and the reference coordinates from IGS SINEX are used to verify the correction of the determined period. The bias of DARW on 9 January 2017 is shown in Figure 8.

Figure 8 shows the detected start time of the orbit maneuvering. The red marks represent the series before the start time, and the blue marks represent the series after the start time. Whether or not orbital maneuvers occur, pseudo-range observations are also closer to the real distance between the station and the satellite, which are not influenced by orbit maneuvering. The biases of the red section are significantly greater than normal conditions because the backward predicted orbit is inaccurate before the start time. It gradually decreases because the backward predicted orbit is gradually closer to the real orbit. From Figure 8, it is obvious that the difference between the real orbit and the backward predicted orbit gradually decreases. The backward predicted orbit is correct after the end time of the orbit maneuver. However, the variation of pseudo-range observations is small during the period of orbit maneuvering. Thus, the biases calculated by the backward orbit reach normal levels at the start time of orbit maneuvering.

**Figure 9:**
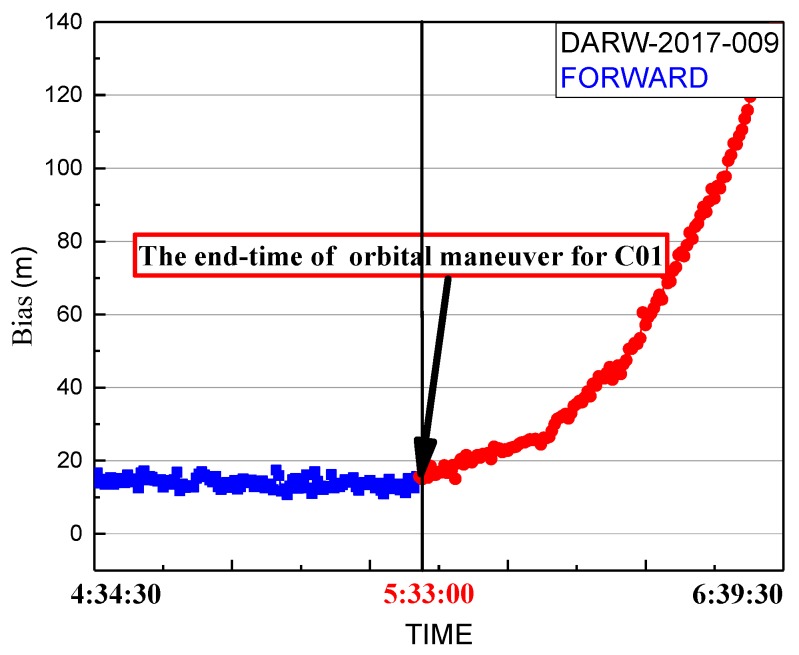
Spatial bias of pseudo-range SPP coordinates for 9 January 2017. Coordinates are calculated by forward predicted orbit using the precise products on the day before orbital maneuvers. The blue line is the series before the end time determined of maneuvering. The red line is the series after the end time determined of maneuvering.

Figure 9 shows the detected end time of orbit maneuvering. The blue marks represent the series before the detected end time, and the red marks represent the series after the detected end time. The biases of the red section are significantly greater than normal conditions because the forward predicted orbit is inaccurate after the end time of maneuvering. It gradually increases because the forward predicted orbit gradually deviates from the real orbit. From Figure 2, it is obvious that the difference between the real orbit and forward predicted orbit gradually increases. The forward predicted orbit is correct before the start time of orbital maneuvering, and the variation of pseudo-range observation is small during the period of orbit maneuvering. Thus, the biases calculated by forward orbit over normal levels mark the end time of orbit maneuvering.

**Figure 10:**
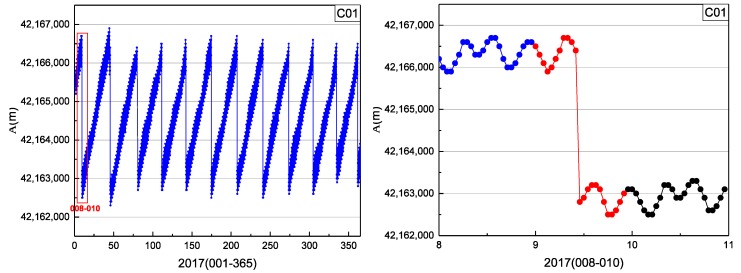
Time series of orbital semimajor axis of C01 in 2017. Graph on the right shows detailed information of the red box (days 008–010) in the plot on the left.

Although the biases of SPP using the data of the nearby reference station are consistent with the orbital maneuver period detection results, it cannot be proved that the biases are only caused by the satellite orbit maneuvers. It is necessary to eliminate the influence of common nonmaneuvering errors. Considering that the orbital semimajor axis will change suddenly before and after the orbital maneuver of the satellite [19], this orbital element could be used as evidence to prove that the orbital maneuver period detected is correct. The time series of orbital semimajor axis from the satellite broadcast ephemeris is shown in Figure 10.

In Figure 10, A is the semimajor axis and the blue points are values of A. The orbital semimajor axis of C01 had several jumps in 2017, caused by the satellite propulsion system changing the original position of the satellite. In the plot on the right, the blue, red, and black marks represent the series of A on days 008, 009, and 010, respectively. Because the position of C01 was changed by orbit maneuvering, the orbital semimajor axis of C01 changed suddenly on days 009.

In summary, it can be considered validated that the period (5:05:30 to 5:33:00) of orbit maneuvering for C01 is correct. The correctness of the method proposed for detecting the orbital maneuver period of GEO satellites is also validated in this study.

#### 3.2.2. Results of Orbital Maneuver Period Detection for IGSO and MEO Satellites

The differences in maneuver period detection between GEO and IGSO/MEO satellites are the station selected and the value of the empirical threshold. MEO satellites cannot be monitored all the time by a single station, and the strategy of selecting stations was adopted from [18]. In order to limit the length of the paper and reduce duplication of text, the results of the IGSO and MEO satellite orbit maneuver period detection and validation are given.

**Figure 11:**
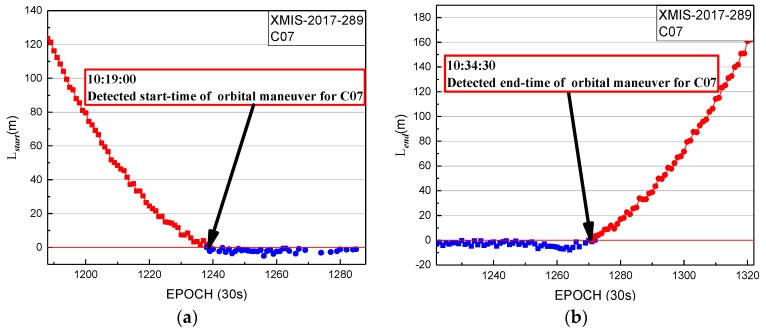
Detection results of XMIS station for C07 on 16 October 2017; (**a**) start time factor of XMIS station for C07 on 16 October 2017; (**b**) end time factor of XMIS station for C07 on 16 October 2017.

In Figure 11, the start time factor series shows a sustained decreasing trend with values greater than 0 until 10:19:00. The start time of orbit maneuvering of C07 determined by the start time factor on 16 October 2017 is 10:19:00. The end time factor series shows a sustained growth trend with values greater than 0 until the end at 10:34:30. The end time of the orbital maneuver of C07 detected by the end time factor on 16 October 2017 is 10:34:30.

**Figure 12:**
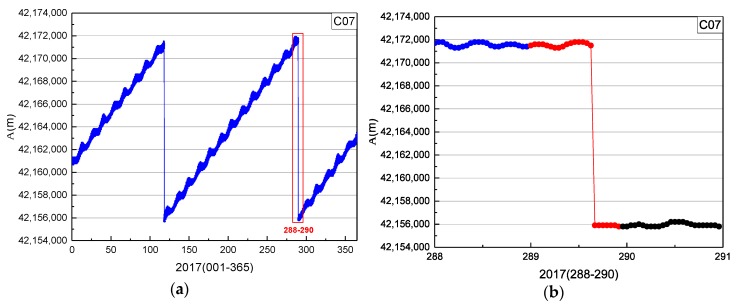
Time series of orbital semimajor axis of C07 in 2017: (**b**) detailed information of the red box (days 288–290) in (**a**).

In Figure 11 and Figure 12, the period of orbit maneuvering for C07 can be determined by the start time and end time factors.

**Figure 13:**
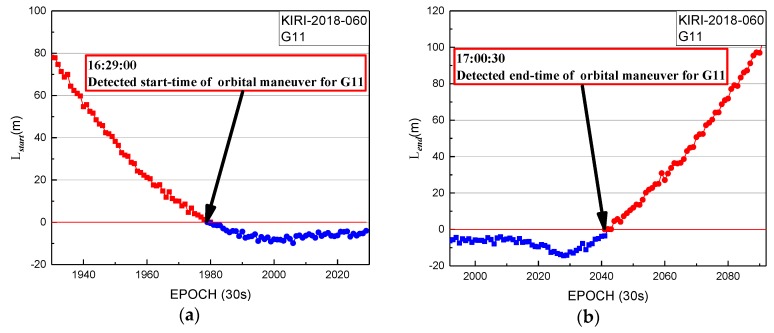
Detection results of KIRI station for G11 on 1 March 2018. (**a**) Start time factor of KIRI station for G11 on 1 March 2018; (**b**) end time factor of KIRI station for G11 on 1 March 2018. Note: the data of station KIRI with a 30 s sampling period from Multi-GNSS Experiment (MGEX) are selected to be analyzed.

In Figure 13, the start time factor series shows a sustained decreasing trend with values greater than 0 until 16:29:00. The start time of orbit maneuvering of G11 determined by the start time factor on 1 March 2018 is 16:29:00. The end time factor series shows a sustained growth trend with values greater than 0 until the end at 17:00:30. The end time of the orbital maneuver of G11 detected by the end time factor on 1 March 2018 is 17:00:30.

**Figure 14:**
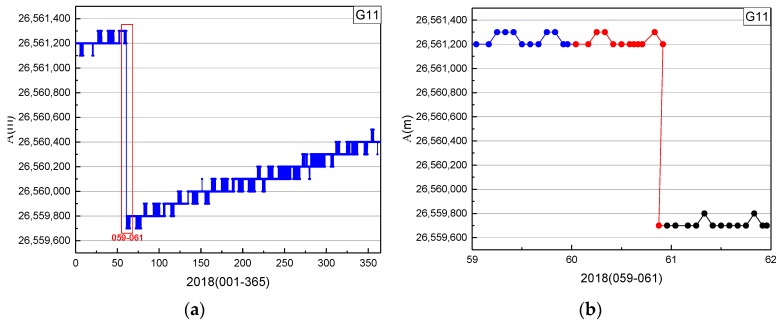
Time series of orbital semimajor axis of G11 in 2018: (**b**) detailed information of the red box (days 059–061) in (**a**).

In Figure 13 and Figure 14, the period of orbit maneuvering for G11 can be determined by the start time and end time factors.

In summary, the orbit maneuvering periods of IGSO and MEO satellites can be detected by the proposed method in this study.

## 4. Discussion and Conclusions

This study proposes an approach to determine periods of orbit maneuvering using publicly broadcast data. The orbit after maneuvering can be calculated by the measured orbit of the day, which supplements the position of satellites for about 7–8 h in the broadcast ephemeris. The observations of MGEX stations, the measured orbits published by CHD, and the coordinates of stations referenced from IGS SINEX were selected to validate the detection method. The results show that periods of orbit maneuvering can be detected accurately for BeiDou GEO and IGSO satellites. In addition, the orbital maneuvering periods of other GNSS MEO satellites can also be determined by this method.

Comparing the method in this study with our previous research methods, referring to [17,18], the first method in previous research methods to detect the start time of orbit maneuvering is based on the mean square unit weight error of SPP technology, which is according to the internal accuracy; the second method in previous research methods detects the start time by the difference of pseudo-range observations and the distance calculated by broadcast ephemeris and station coordinates. The two methods can detect only one orbit switching time because the orbit after maneuvering is inaccurate from broadcast ephemeris. In addition, the variation of pseudo-range observations is small due to the orbital maneuvers, which could not be considered in previous research. It causes the detected start time of orbit maneuvering to have some latency, but the average time of orbital maneuver difference between the detected start time and the marked started time of the broadcast ephemeris is 91 min in 2017. It means that the proposed method can extend the usable observations for a few hours when the satellite is maneuvered for users. Thus, the method proposed in this paper can detect the start time and end time of orbit maneuvering more accurately, and the period of orbit maneuvering can be determined. The result of period detection for orbit maneuvering provides important reference information for precision orbit and clock offset determination when carrying out satellite maneuvers. In our next research, the detection results of orbit maneuvering of this study will be used to determine the orbit and clock offset in order to improve the integrity of precision orbit and clock products.

## Figures and Tables

**Table 1:** Thresholds of orbital maneuver detection for XMIS in 2017 (unit: meter).

PRN	C01	C02	C03	C04	C05	C06	C07	C08	C09	C10	C11	C12	C13	C14
$T_{Max}$	10.7	2.9	5.2	4.8	5.2	4.8	7.8	6.4	3.6	2.9	8.8	5.9	19.2	3.6
